# Promoting Data Sharing: The Moral Obligations of Public Funding Agencies

**DOI:** 10.1007/s11948-024-00491-3

**Published:** 2024-08-06

**Authors:** Christian Wendelborn, Michael Anger, Christoph Schickhardt

**Affiliations:** 1 Section for Translational Medical Ethics, German Cancer Research Center (DKFZ), National Center for Tumor Diseases (NCT) Heidelberg, Heidelberg, Germany; 2 Present Address: University of Konstanz, Konstanz, Germany

**Keywords:** Data sharing, Epistemic integrity, Funding agencies, Moral obligations, Research integrity, Scientific progress, Scientific freedom, Social value

## Abstract

Sharing research data has great potential to benefit science and society. However, data sharing is still not common practice. Since public research funding agencies have a particular impact on research and researchers, the question arises: Are public funding agencies morally obligated to promote data sharing? We argue from a research ethics perspective that public funding agencies have several *pro tanto* obligations requiring them to promote data sharing. However, there are also *pro tanto* obligations that speak against promoting data sharing in general as well as with regard to particular instruments of such promotion. We examine and weigh these obligations and conclude that *all things considered* funders ought to promote the sharing of data. Even the instrument of mandatory data sharing policies can be justified under certain conditions.

## Introduction

The potential benefits of sharing research data for science and society have been widely acknowledged and emphasised. Some disciplines or sub-disciplines have a longstanding tradition and well established practices of data sharing, for instance, astrophysics, climate research and biomedical genomic research. However, despite various efforts to promote and encourage data sharing, for instance by scientific journals, it is still not common practice in most fields of the sciences. As public funding agencies have considerable influence on both the scientific communities as well as the individual researchers, the question arises whether they are morally obligated to promote data sharing. In order to answer this question, we examine the following more specific three questions from the perspective of research ethics:

1. Do public funders have general *pro tanto* moral obligations that require them to promote data sharing?
2. Do public funders have general *pro tanto* moral obligations that speak against promoting data sharing?
3. What *pro tanto* moral obligations have to be considered in the particular case of using *mandatory* data sharing policies, i.e., policies that *require* researchers to share data?

Answering these questions is a desideratum of (bio)ethical research on issues of data sharing. Although it is stated that individual researchers have a scientific responsibility (Bauchner et al., 2016; Fischer & Zigmond, 2010) and even a moral obligation to share data (Schickhardt et al., 2016), the moral responsibilities and obligations of public funding agencies in matters of data sharing have not been discussed systematically and explicitly from the perspective of research ethics. While it is common to postulate that funders “should” encourage data sharing or that it is their “responsibility” to do so, we want to carry out an in-depth ethical analysis of funders’ moral obligations. In doing so, we also contribute to an analysis of what funders are *generally* morally obligated to – another question that has thus far been rather neglected in research ethics and discussed primarily in terms of priority-setting and with regard to the general obligation to benefit society (Pierson & Millum, 2018; Pratt & Hyder, 2017, 2019). Thus, we will provide a broader analysis of general moral obligations of funders and evaluate what they imply with regard to promoting data sharing in particular.

We proceed as follows: After some preliminary remarks in Sect. "Preliminary Remarks", we provide a brief review of empirical data on the current status quo of data sharing in Sect. "The Current State of Data Sharing and of Promoting Data Sharing". In Sect. "The Moral Obligations of Funders and the Promotion of Data Sharing", we set out that funders have three general moral *pro tanto* obligations that require them to promote data sharing. In Sect. "Further Relevant Moral Obligations", we examine two *pro tanto* obligations that both speak in favour of *and* against promoting data sharing. We conclude Sect. "Further Relevant Moral Obligations" by weighing all *pro tanto* obligations. In Sect. "Mandatory Data Sharing Policies and Academic Freedom", we ethically assess the specific instrument of promoting data sharing by way of mandatory policies with regards to academic freedom. We conclude and summarise our arguments in Sect. "Summary and Conclusion".

## Preliminary Remarks

1. In the following, we use the term “research data” and “data” as referring to *digital data* that is collected and/or generated during a research project. We use the term “data sharing” as referring to the act of making data available for other researchers – either for the purpose of transparency of studies and replication of published research results or for the purpose of other researchers using the data for their own research questions and projects (secondary research use).^1^ Data sharing is increasingly supposed to meet the requirements of the FAIR principles, i.e., data should be findable, accessible, interoperable and re-usable (Wilkinson et al., 2016). Data can be shared in various ways, for example via open access or restricted or controlled access, and by using Data Use and Access Committees or data sharing licenses.^2^ Restricted or controlled access comes, for instance, with additional data protection requirements when personal data are involved. Data sharing activities (and data sharing policies by funders) must comply with the applicable local laws and regulations. In EU countries, the possibilities for international sharing of non-anonymous data are dependent on the EU GDPR, making personal data sharing difficult between EU countries and the US, for example. As to legal challenges to international data sharing raised by local laws, there are possible legal approaches (contracts) and technical solutions such as code-to-data approaches, when the data remains at the location of the data producer or the repository and is only analysed there on behalf of the other researcher.^3^
2. We define public funding agencies, following the European Commission Joint Research Centre (2017), as organisational entities that distribute public funding for research on behalf of either regional, national, or transnational governments. The definition covers both i) funding agencies operating at arm’s length from the public administration and enjoying relative autonomy from the government and ii) ministries and offices within the government that fund research projects. The definition comprises of centralised and non-discipline specific agencies such as the German Research Foundation (Deutsche Forschungsgemeinschaft), de-centralised and discipline specific agencies such as the National Institutes of Health in the US or the UK Research Councils, as well as international funding agencies and bodies such as the European Commission. When we speak of research funding, we refer to funders who grant funds to individual researchers or groups of researchers (collaborative projects or research consortia). Against the background of the existing organisation of the (academic) science system with its systematic competition between researchers and the importance of scientific publications, we assume that funded researchers use the funding to seek and publish new findings and that they do so in a somehow exclusive way that does not involve the immediate disclosure of all data and results. The tendencies of competition, exclusive use of data and the pursuit of (more or less) exclusive first scientific publications of previously unknown research results are the reasons why funders' policies on sharing research data and overcoming data secrecy are important, at least at some point in the project and research cycle. Traditionally, research projects funded in this way tend to be hypothesis driven. However, as research methods, the nature of projects and the associated research funding evolve rapidly and potentially change in the era of Big Data and AI, the boundaries are blurring, and some things may change. There might be more scientific community-led research projects that are designed to be less exclusive and competitive, with community participation, immediate disclosure, and data sharing at the forefront from the start. A historical example is the Human Genome Project. Funding of such community-led research projects is not the focus of our paper, but community-led research is worth mentioning and discussing in further research.
3. As public funders are public (or even state) institutions and spend public money that they receive from the government, their moral obligations are related to their public and therefore political character. Our analysis of the moral obligations assumes a liberal-democratic and rights-based normative-ethical framework. To put it simply, public institutions are normatively conceived as "by the people, for the people and of the people", and citizens, including researchers, have fundamental liberal rights vis-à-vis the state and public institutions, especially negative rights that protect them from state interference. These moral rights, which play an important role in our analysis, include academic freedom and the rights to privacy and informational self-determination.
4. We confine our analysis in this article only to the promotion of data sharing within academic science and exclude the question of the promotion of data sharing from publicly funded academic science with private for-profit companies.
5. We do not limit our argument to funders that focus on a particular scientific discipline (for instance, biomedical funders), as we believe that the *pro tanto* obligations we will attribute to funders do not depend on the specific characteristics of particular scientific disciplines. However, we think that when applying our framework in practice, context factors that depend on the features of a certain discipline or a specific research project need to be taken into account.
6. Some of the following arguments for the moral *pro tanto* obligations of public funders can be translated *mutatis mutandis* to private funders, but not all of them can. Particularly those arguments that refer to the special status of public funders as public institutions that spend public money and have particular responsibilities towards the public and the rights of citizens cannot be applied to private funders. The obligations of private funders call for a separate analysis in a separate paper.
7. This paper presents an ethical analysis of the *moral* obligations of funders and is not concerned with legal rights and obligations that pertain to funders in a particular national state or legal area such as the European Union. We assume that the moral obligations presented below do not conflict with the legal requirements of (public) funders in any legal context. However, our claims that funders have a moral obligation to promote data sharing and that they should also implement mandatory data sharing policies under certain circumstances have implications for the revision of (templates for) future legally binding funding contracts between funders and funded researchers. In this respect our ethical analysis has legal implications.
8. We take a *pro tanto* obligation as an obligation that has some weight in determining what an actor morally ought to do *all things considered* (Dabbagh, 2018). Suppose I promise my friend to visit her tonight; however, my daughter is sick, and I ought to stay with her. I have then two *pro tanto* obligations that prescribe conflicting actions. To find out what I am obligated to do *all things considered*, I must find out which of the two obligations weighs heavier.^4^ Therefore, when we examine *pro tanto* obligations that require the promotion of data sharing, these obligations must be weighed against other *pro tanto* obligations that speak against such promotion.

## The Current State of Data Sharing and of Promoting Data Sharing

As to the current state of data sharing, there are differences across scientific disciplines (Tedersoo et al., 2021a, 2021b). Some disciplines, such as astrophysics, climate research or genomic research, have a long history of data sharing. For instance, genomics research paved the way with the important and pioneering Fort Lauderdale (Fort Lauderdale Agreement, 2003) and Bermuda principles (First International Strategy Meeting on Human Genome Sequencing, 1996) on data sharing (Kaye et al., 2009) within the revolutionary and community driven Human Genome Project and has created a genomic commons, i.e., openly available data bases for genetic and genomic driven biomedical research (Contreras & Knoppers, 2018; National Cancer Institute; National Library of Medicine). With the exception of some more advanced scientific disciplines or sub-disciplines, the sharing of research data for purposes of transparency and secondary use still remains the exception rather than the norm in most fields and disciplines of the sciences (Danchev et al., 2021; Gabelica et al., 2022; Naudet et al., 2021; Ohmann et al., 2021; Thelwall et al., 2020; Watson, 2022; Strcic et al., 2022; Gorman, 2020; Towse et al., 2021). While there is an increased awareness of the benefits and importance of data sharing in all of the sciences and although various initiatives of funders and journals promote data sharing, for instance through data sharing policies, data sharing is still not common practice. Several studies report rather low rates of compliance with data sharing expectations or requirements of funders and journals (Couture et al., 2018; Federer et al., 2018; Gabelica et al., 2022; Naudet et al., 2018, 2021; Danchev et al., 2021). Studies also report a gap between high in-principle support for data sharing, and low in-practice intention (Tan et al., 2021).

It is frequently emphasised that funders should improve and intensify their current efforts to promote data sharing. Some see the need to create incentives, for example by including a record of past data sharing as an additional criterion for the reviews of grant applications (Perrier et al., 2020; Terry et al., 2018). Since the majority of funders’ data sharing policies do not strictly require the sharing of data (Ohmann et al., 2021), some authors call for stronger policies with strict requirements for data sharing (Couture et al., 2018; Naudet et al., 2021; Ohmann et al., 2021; Sim et al., 2020; Stewart et al., 2022; Tedersoo et al., 2021a, 2021b)^5^ and contest the lack of monitoring and enforcing compliance (Couture et al., 2018; Kozlov, 2022). However, as a series of interviews shows, funders struggle to implement data sharing requirements, incentives, monitoring, and sanctions for non-compliance for various reasons (Anger et al., 2022, 2024).

In consideration of the foregoing and from the perspective of research ethics, the question arises whether public funders are *morally* obligated to promote data sharing. To answer this question, in the next section we set out a description and analysis of funders' general moral obligations and their relevance for data sharing.

## The Moral Obligations of Funders and the Promotion of Data Sharing

We will argue that funding agencies have several general moral *pro tanto* obligations requiring them to promote data sharing: The obligation to benefit society, the obligation to promote scientific progress as such and the obligation to promote scientific integrity. Our methodological approach consists of first introducing and explaining the individual moral obligations in order to then briefly justify them with reference to plausible and, for the most part, generally shared fundamental considerations, values or norms.

### The Obligation to Benefit Society

Publicly funded research should benefit society, or, as it is sometimes put, it should have social value.^6^ As a requirement for public funders, this means funders should base their decisions on considerations of social value. Barsdorf and Millum (2017) argue that funders ought to consider the social value in particular in their priority-setting, i.e., when setting goals and priorities for the research they fund. We extend the obligation to promote social value to all decisions and actions of public funding agencies.^7^ Benefitting society or social value is sometimes conceptualised in terms of well-being. The concept of well-being is notoriously controversial in philosophy (as it relates to the complicated and controversial topic of the “good life”). In research ethics, the benefits at stake in the social value obligation are sometimes framed more pragmatically, for example when Resnik (2018b) (following Kitcher 2001) states that benefits are “practical applications in technology, industry, medicine, engineering, criminal justice, the military, and public policy”, and that these applications “can also produce economic growth and prosperity”. We limit our conception of social value (benefit) to a more basic understanding (which does not include potentially problematic or controversial elements such as military and economic growth): We understand it in terms of the basic goods of health and wealth (housing, food, employment, income, etc.), infrastructure development (for communications, travel, etc.), and environmental protection (as natural resources).

What are the justifying reasons for this obligation? First of all, it must be pointed out that the obligation can be understood in different ways, depending on whether the population to be benefited is the local or the global population. Barsdorf and Millum (2017), for instance, argue that for health research the social value obligation of funders is towards the global and not the local (national) population of the funders’ country. In the literature, this question (local vs. global) is controversial. In general, the controversial positions on this question also depend on the justification one is willing to accept for the obligation. For instance, if one justifies the obligation as owed to the citizens as tax payers who finance the state and the public funder via taxes, then it is rather obvious to understand social value as benefit for the national tax paying population. In contrast, if one considers the social value obligation of funders as owed to all humans all over the world, it suggests itself to understand the social value broadly in terms of global benefit for all humans. Such a global understanding of the social value obligation could be justified with considerations of beneficence towards every human being or with a universalistic-egalitarian account of human rights. Global understandings of the obligation are likely to give priority to poor populations of the global South. We deem a combination of a local and a global understanding as being the most plausible one: funders have a primary obligation to foster social value on the national level, and an additional (weaker) social value obligation on a global level. But even this combined view raises questions and cannot be elaborated here. Most importantly for the purpose of our paper, we believe that the question concerning the understanding of the social value obligation(s) of funders (towards national vs global population or both) is not relevant for our question about the promotion of data sharing by funders. At first glance it might seem that a local reading of the social value obligation suggests that funders should promote sharing of research data only among local/national researchers. However, the contrary is much more plausible, at least for the academic sciences. Most fields of modern academic scientific research are international endeavours and advancements are achieved through multiple and interacting contributions from scientists from different countries. In most disciplines, there is no such thing as a „national current state of scientific progress “. As for sharing research data from the academic and publicly financed sciences with private for-profit companies, it might be plausible to assume that sharing data only with national companies is more likely to benefit the national population than sharing data with for-profit companies from abroad. However, this assumption can also be challenged, for example, in light of the rapid and effective development of vaccines during the covid pandemic. Most importantly, the sharing of research data from the publicly funded academic sciences with private for-profit companies is a very specific topic that we do not address in this paper.^8^ As far as sharing of research data between academic researchers is concerned, it is plausible to assume: The more data are shared on a national and international level, and the more science advances – which in almost all scientific disciplines occurs as an international advancement -, the more likely national populations will benefit.

A last and more specific reason for funders’ obligation to foster social benefit is the following, which applies only to research involving humans or animals: If funders fund research that exposes animals and humans to risks and burdens, the funding can only be justified if the potential benefits for society are maximised (National Commission for the Protection of Human Subjects of Biomedical & Behavioral Research, 1978; World Medical Association, 2013).^9^

The concept of social value refers to (classical and much debated) questions of distributive justice: Of all persons concerned, *who* should benefit *how much*? Following Barsdorf and Millum, we think the obligation to benefit society, i.e., the social value obligation, should be understood according to a prioritarian account of social value. On a prioritarian account, benefits should be distributed such that the distribution (expectedly) maximises “weighted well-being” (or in our terms “weighted social benefit”), i.e. the well-being of the worse off gets some priority in the distribution of benefits.

Let's put this in the following proposition and call it *the social value obligation* for public funders:

Funders have a *pro tanto* obligation to align their decisions and actions in such a way that the research they fund maximises weighted social benefit.^10^

Now, what is the relevance of the social value obligation for matters of promoting data sharing? We develop our answer step by step:

*First step*. Data sharing has the potential to optimise research in terms of i) progressiveness, ii) cost and iii) quality (Fischer & Zigmond, 2010; Sardanelli et al., 2018). Ad i) The sharing of research data accelerates research, enables more cooperation and collaboration between researchers and disciplines, allows for the integration and pooling of data from disparate sources into large data sets, and bears the potential for innovative research, meta-analyses and new lines of inquiry that can lead to better diagnoses and treatments. Ad ii) It reduces costs and is efficient as reusing the data increases the value of the initial investment. Ad iii) It allows research findings to be verified or reproduced based on the original data and thus increases the quality of research and potentially reduces “research waste” (i.e., research with questionably quality).

*Second step*. Given this efficiency-, quality- and progress-enhancing potential of data sharing, it is rational to assume that the following holds true: *A world in which funded researchers share their data is better in terms of social value than a world in which funded researchers do not share their data.* Notice that this holds true only under the following conditions: a) Funders must set research funding priorities according to the social value obligation. It is plausible to assume that only the sharing of data from research projects that were selected according to the right priorities (expectedly) maximises weighted social benefit. b) The funding of secondary use and decisions on data access for secondary use must be aligned to the social value obligation as well.^11^

*Third step*. From the claim that a world in which funded researchers share their data is better in terms of social value it does not directly follow that funding agencies are obligated to promote a world in which researchers share their data, for two reasons:

1. If there are alternative actions than promoting data sharing that lead to a larger increase in weighed social benefit and that cannot (for cost or other reasons) be taken together with promoting data sharing, then these alternative actions should be taken. For instance, perhaps an initiative to promote translational biomedical research increases weighed social benefit more than the promotion of data sharing and the funder's budget can only finance one of the two initiatives.
2. Realising a world in which researchers share data comes with costs, for instance for warranting long-term storage and data availability or for incentivising data sharing. Hence, it may be that the means to realise a data sharing-world are so costly that they cancel out the benefits data sharing brings, so that realising this world does not maximise weighted social benefit and ought not to be done.

However, we think that both possibilities are very unlikely. Ad 1. We deem it highly unlikely that there are alternatives that are incompatible with promoting data sharing and more efficient in terms of social value. Ad 2. We think that the means to realise a world in which researchers share their data are not so costly that they cancel out the benefits. For instance, incentivising data sharing or making data sharing mandatory are means that can be expected to promote data sharing without being too costly.^12^

Therefore, we conclude: To fulfil the social value obligation, funders *pro tanto* ought to promote data sharing.^13^

This conclusion leaves open which specific means of promotion funders are required to take. Since there are many ways of promoting data sharing, some of which are cheaper, some of which are more effective, the social value obligation – in principle – requires a specific means of promotion. For example, incentivising data sharing (for instance, through data sharing prizes or other forms of recognition) might be cheaper but less effective, whereas mandatory policies in combination with monitoring and sanctioning might be more expensive but lead to a greater extent of data sharing. It is an empirical question which of these different means (or combination of means) maximises weighted social benefit (for each situation of each individual funder). We cannot answer this question here. For now, we confine ourselves to the conclusion that the social value-obligation *pro tanto* requires funders to promote data sharing and leave it open which specific means of promotion they ought to apply.^14^

### The Obligation to Promote Scientific Progress

In addition to the social value obligation, public funding agencies have a *pro tanto* obligation to promote scientific progress. Since scientific progress is likely to increase the social value of scientific research, one reason for funders’ obligation to promote scientific progress is the already discussed social value obligation. However, beyond social value there are also other reasons for the obligation to promote scientific progress and these reasons ground an independent obligation to promote scientific progress. In the following, we focus on these reasons that justify the obligation to foster scientific progress independently from social value.

1. In democratic countries, public funders have an obligation to promote scientific progress, i.e., the growth of (significant) scientific knowledge and understanding,^15^ because it is their mandate to support a science system that is geared towards producing scientific knowledge (independently of considerations of social benefits). In most democratic countries this mandate is institutionalised on a constitutional level. In this sense, funders owe this obligation to the (democratic) public and the citizens.
2. There is a set of further reasons that justify the obligation of funders to support the scientific system and foster scientific progress with considerations of the value of scientific knowledge and progress. The value of science and scientific progress touches on complex questions about whether knowledge is valuable in itself and/or (only) insofar as it is somehow conducive to realising other values or ends. We do not want to take a position here on the hotly contested question about whether scientific knowledge (or progress) are intrinsically valuable (end in itself).^16^ We just want to point to the aspects of knowledge that make knowledge instrumentally valuable apart from its instrumental value for the benefits of society. i) Scientific knowledge can be instrumentally valuable when it satisfies “human curiosity” (Kitcher, 2001) and the desire for a practically disinterested understanding of the natural world. ii) Scientific knowledge is a precondition and a contributory factor for the ability and freedom of “pursuing our own good in our own way” (Mill, 2008) and making reflective decisions about the goals of our own lives. By expanding our understanding of the world and our place in it, scientific progress can contribute to the exercise of this elementary freedom and can thus be seen as valuable for a self-governed and autonomous life (Kitcher, 2001; Wilholt, 2012). iii) scientific knowledge and progress is valuable for a functioning democracy insofar as (growth of) knowledge is a requirement for processes of informed deliberation, opinion-forming and decision-making (Brown & Guston, 2009). Now, this set of three reasons (i-iii) could be understood as reflecting not only the values and interests of the citizens (or tax payers) of the funder’s country, but also the values and interests of all people all over the world. Although it is plausible to some extent that the three reasons also reflect values or interests of people around the world, we do not think that this can establish a relationship in terms of strong moral rights and obligations between the global population and the local funder. Due to the rather loose relationship between persons in each country of the world on the one hand and the local state and funder on the other hand, only rather weak reasons for funders to promote scientific progress could result from the global understanding of the three reasons.
3. So far, we have argued that the obligation of funders to promote scientific progress is primarily owed to the public and the citizens (and rather weakly to the global population). But of course the question arises whether funders owe the promotion of scientific progress also to scientists or the scientific community. We think that this is the case. Scientists have the professional obligation to strive for scientific knowledge and progress. To fulfil this professional obligation, they depend on the scientific system in which funders play an important role. Scientists need a functional system that is designed to enable and promote scientific progress. Therefore, it is plausible that funders owe the obligation to promote scientific progress to the scientists as well.

We take the *scientific progress obligation* as follows:

Funders have a *pro tanto* obligation to align their decisions and actions such that the research they fund maximises scientific progress.

What relevance does this obligation have when discussing funders’ role in promoting data sharing? First and in general terms, this obligation to maximise scientific progress does not necessarily require funders to exercise intensive control and strong intervention in science. Keeping funders largely out of the methodological and content-related decisions of researchers is plausibly conducive to a functioning and progress-making scientific system. However, specific measures or interventions on the part of funders (for instance through policies) might have the potential to promote scientific progress. The promotion of data sharing plausibly is such an intervention: As we argued in Sect. "The Obligation to Benefit Society", a scientific system in which researchers share their data can be expected to be a more efficient, effective, and innovative scientific system, and this means that it is also a better system in terms of scientific progress than a system in which researchers do not share data. Funders can contribute to realising such a system through various means (such as, for instance, data sharing policies) and thus promoting scientific progress.

However, as it is the case of the social value obligation as well, it does not follow directly that funders are obligated to promote data sharing. This depends on whether there are other means than promoting data sharing which are more conducive to scientific progress (and which cannot be taken together with the promotion of data sharing). Again (as with the social value obligation), this is an empirical question that we cannot answer here. Nonetheless, we think it is plausible to assume that promoting data sharing is an effective and efficient means to promoting scientific progress and that it is rather unlikely there are other more efficient and effective actions or means, which, at the same time, are incompatible (for cost or other reasons) with the promotion of data sharing.^17^

Accordingly, to fulfil their moral obligation to use the resources at their disposal to maximise scientific progress requires them to promote data sharing.

### The Obligation to Promote the Epistemic Integrity of Research

Public funding agencies have an obligation to promote the integrity of the research they fund—a view which is widely held (Bouter, 2016, 2018, 2020; Mejlgaard et al., 2020; Titus & Bosch, 2010), but not systematically developed and justified. To give a more detailed account of this obligation, we start with clarifying the concept of research integrity.

Research integrity relates to a set of professional norms and obligations that morally regulate and prescribe how researchers ought to conduct research. These norms and obligations can be differentiated between *epistemic and socio-moral norms and obligations*.^18^ Epistemic norms or obligations are grounded in the goals or nature of science (Resnik, 1998), i.e., (roughly) the goals to obtain knowledge and understanding through reliable methods of inquiry. These obligations prohibit misconduct that is problematic from the point of view of epistemic rationality*.* Epistemic obligations are, for instance, the obligation not to fabricate, falsify, or misrepresent data. Epistemic obligations form what one might call *epistemic research integrity*. We take epistemic research integrity to be mainly about avoiding practices that lead to deception, inaccuracy, and imprecision in research and (the presentation) of research results. We thus follow Winter and Kosolosky (2013), who explicate the notion of epistemic research integrity by drawing on the property of deceptiveness and “define the epistemic integrity of a practice as a function of the degree to which the statements resulting from this practice are deceptive.”

*Socio-moral* obligations result from the fact that research can negatively affect the rights and interests of individuals or groups outside science. Such non-epistemic obligations take into account general responsibilities and potential effects of science for society and humanity and comprises, for example, obligations to obtain consent and to minimise risks for participants and third parties. These socio-moral obligations constitute what one might call *socio-moral research integrity*.

In the following, we focus only on *epistemic research integrity* and investigate whether funders’ obligation to promote epistemic research integrity implies that they ought to promote data sharing. We briefly address the relationship between data sharing and socio-moral research integrity in Sect. "Further Relevant Moral Obligations".

The promotion of epistemic research integrity is required by the two above mentioned obligations of funders to promote social value and scientific progress since epistemic integrity arguably furthers social value and scientific progress or is even a prerequisite for them. Now, the goal of this section is to show that there are reasons independent from social value and scientific progress that ground or justify an obligation of funders to maximize epistemic research integrity. There are two reasons for this as an independent obligation in its own right:

Funders should promote the epistemic integrity of research for two reasons. 1. As public funders are either governmental institutions or at least spend public money, they should ensure that the activities they finance abide by *professional* norms and standards. Funders are not supposed to spend public money on activities where “anything goes” but rather fund activities and work that are *lege artis*. This is owed to the citizens and taxpayers and required by the recognition of the value of a rules-based scientific system. 2. Funders must guarantee a fair and rule-based research environment and competition. This is primarily owed to the scientists, among other things, to protect the honest and *bona fide* researchers against unfair and dishonest competitors.

In the following, we take the obligation of funders to promote epistemic research integrity as follows:

Funders have a *pro tanto* obligation to align their decisions and actions such that they maximise the epistemic integrity of research.

What does the obligation to promote epistemic research integrity imply for the question of whether funders ought to promote data sharing? To answer this question, we must investigate whether data sharing is required by epistemic research integrity.

To begin, we must differentiate between two different perspectives on epistemic research integrity. One perspective can be labelled as *normative*-*philosophical* and takes research integrity as a set of philosophically justified norms. The other perspective can be labelled as the *community consensus* perspective and takes research integrity as a set of norms that are agreed on and prescribed by the scientific community and that are codified in statements and codes of conduct by scientific societies and associations. These two perspectives usually do not display great discrepancies in terms of concrete norms of research integrity, but in principle they are not necessarily congruent. For reasons of space, we cannot give a systematic answer to the question of which of the two perspectives takes normative priority when they have conflicting norms and prescriptions. However, in the following we first examine the relationship between epistemic integrity and data sharing from a philosophical perspective and then describe how this relationship is treated in relevant codes of conduct and guidelines on research integrity. We will show that the two perspectives converge to some extent, and where they do not clearly converge, we will explain what this means for funders. We will do this in turn for data sharing for transparency (A.) and data sharing for secondary use (B.).^19^

#### A. Epistemic Integrity and Data Sharing for Transparency

*1. Philosophical perspective*: Philosophers of science consider practices that enable “each scientist to scrutinize the work of others in his field, to verify and replicate results [and that make] it more likely that flaws will be uncovered” (Haack, 2007) to be prescribed by an important epistemic norm. The pertinent norm here is what David Resnik calls the “principle of openness” (Resnik, 1998) or what Susan Haack calls the epistemic norm of “evidence-sharing” (Haack, 2007). According to this understanding, practices of evidence-sharing enable collective efforts of communicating, reviewing, critiquing, and reproducing the evidence claimed by researchers as supporting their scientific claims and research results, i.e., evidence “which includes the methodology applied, the data acquired, and the process of methodology implementation, data analysis and outcome interpretation” (Munafò et al., 2017).^20^ The sharing of evidence is a necessary condition for science as rational communication and argumentation and a requirement for efforts of reviewing and assessing scientific claims. Evidence-sharing can thus be understood as part of an organized skepticism^21^ that increases the credibility of scientific claims and characterises (the ideal of) modern science as a specific social and cooperative enterprise. Following Winter and Koslovsky (2013), the principle of openness and the norm of evidence-sharing can be understood as prescribing practices that prevent and guard against deceptiveness.

One of these practices is arguably *data transparency, i.e., transparency with respect to data on which an already published scientific paper is based.* We want to explicate at least two reasons for why data transparency is an important norm of evidence-sharing and openness.

i) *Data sharing as a prerequisite for replication*. It is widely agreed that replication studies have epistemic value and are an essential and important part of scientific practice at least in a substantial part of the quantitative empirical sciences. Even those who caution against the crisis narrative in connection with failed replications or even doubt the epistemic value of replications for all disciplines (Leonelli, 2018) agree with this proposition. However, a precondition and minimal requirement for conducting replication studies is that the original studies can be (computationally or analytically) *reproduced*, that is, the published findings can be reproduced when the reported analyses are repeated upon the raw data (Hardwicke et al., 2021; Nuijten et al., 2018; Peels & Bouter, 2021). If a result cannot be reproduced, there is no need to even attempt a replication – since something with the analysis or the data must have gone wrong. Therefore, if we agree that efforts to replicate should be enabled and encouraged (due to its important epistemic value for research), then we must also recognise the importance of data transparency.
ii) *Data sharing as means for preventing and detecting breaches of epistemic integrity.* Although the empirical evidence about the prevalence of scientific misconduct and questionable research practices (QRP) should be handled with care, studies suggest that it is non-negligible. For instance, a survey among researchers in The Netherlands found that “over the last three years one in two researchers engaged frequently in at least one QRP, while one in twelve reported having falsified or fabricated their research at least once” – with the highest prevalence estimate for fabrication and falsification in the life and medical sciences (Gopalakrishna et al., 2022). Similarly worrisome results with regard to different forms of questionable research practices or misconduct are reported in (Boutron & Ravaud, 2018; John et al., 2012; Kaiser et al., 2021).^22^ Additionally, we think that it is not entirely unreasonable to assume that the widespread lack of transparency (particularly the much-reported difficulties of obtaining data even after personal requests) is at least somewhat indicative of a non-negligible prevalence of scientific misconduct and questionable research (data) practices.^23^

The possibility of keeping data opaque enables misconduct or at least makes it more difficult to detect it. As data transparency makes it easier to detect (at least some forms of) fraud and questionable research practices and can function as a deterrent (Fischer & Zigmond, 2010; Gopalakrishna et al., 2022; Hedrick, 1988; Winter & Kosolosky, 2013), we argue that data sharing for transparency can help prevent and detect unethical scientific practices.

Since data transparency is a prerequisite for reproducibility and a means for preventing and detecting misconduct and questionable research practices, we conclude that there are good (normative-philosophical) arguments for taking data sharing for transparency as an important requirement of epistemic research integrity.

*2. The community consensus perspective:* The scientific community also sees data sharing as an important part of epistemic integrity (All European Academies ALLEA, 2017; Deutsche Forschungsgemeinschaft (DFG), 2019; Kretser et al., 2019; National Academies Press (US), 2017; Netherlands Code of Conduct for Research Integrity, 2018; Resnik & Shamoo, 2011; World Conference on Research Integrity, 2010). However, most of these guidelines and codes of conduct do not explicitly differentiate between epistemic and socio-moral integrity of research and many do not clearly differentiate between the purposes of data sharing (i.e., the purposes of transparency and secondary use). Therefore, we must deduce from the context what the respective statements refer to. We cannot do this in a systematic way here. But our impression is that many documents emphasise the values of transparency and honesty and explicitly or implicitly refer to these values when they state the importance of data sharing for research integrity. It thus seems there is a (international and trans-disciplinary) consensus that data sharing for purposes of transparency is a part of epistemic integrity. For example, the Netherland Code of Conduct explicitly connects data availability with the value of transparency, and the German DFG also explicitly refers to data sharing for the purpose of confirmability (“Nachvollziehbarkeit”).

Hence, both perspectives—the normative-philosophical and the community consensus perspectives—support the proposition that data sharing for transparency is an important component of epistemic research integrity.

#### B. Epistemic Integrity and Data Sharing for Secondary Use

1. *Philosophical perspective*: While data sharing for transparency clearly falls within the scope of epistemic research integrity, the same cannot be said about data sharing for secondary use. Since we follow Winter and Kosolosky (2013) and “define the epistemic integrity of a practice as a function of the degree to which the statements resulting from this practice are deceptive”, we believe that data sharing for secondary use is not part of epistemic research integrity. Although one might argue that secondary use of data has the potential to correct for misleading or deceptive statements from original studies, we think that the main importance of sharing data for secondary use is that it promotes scientific progress and social value. Data sharing for secondary use is of rather secondary importance when it comes to correcting misleading scientific statements or results. It does not seem to be a strict requirement of epistemic integrity but more of a supererogatory practice. Therefore, from a philosophical perspective, the promotion of data sharing for secondary use is not required by the obligation to promote epistemic research integrity
2. *Community consensus perspective*: Only a few guidelines and codes of conduct explicitly state that data sharing for secondary use is a requirement of research integrity (for instance, DFG, 2019). Many do not mention data sharing for secondary use explicitly, and some do not even seem to consider it implicitly. Thus, there does not appear to be a clear and unambiguous international consensus on the relationship between data sharing for secondary use and epistemic integrity. And since most of these documents do not differentiate explicitly between epistemic and socio-moral integrity, it is not clear whether data sharing for secondary use is considered as important from an *epistemic* perspective or from a non-epistemic, *socio-moral* perspective.^24^
  Therefore, from a community consensus perspective there is no clear consensus that data sharing for secondary use is a requirement of (epistemic) research integrity. From this perspective then, the obligation of funders to promote epistemic research integrity does not require the promotion of data sharing for secondary use. However, if there are specific disciplinary or national communities that explicitly take data sharing for secondary use as part of research integrity, those funders for whom this consensus is pertinent might have a reason to promote this kind of data sharing with reference to the obligation to promote research integrity. This holds true even though from a philosophical perspective data sharing for secondary use is not a part of epistemic research integrity: If the pertinent community takes data sharing for secondary use as part of (epistemic) integrity, funders might take this as a reason to promote it.^25^

Therefore, and to conclude this whole Sect. "The Obligation to Promote the Epistemic Integrity of Research" about research integrity: Since funders have the obligation to promote epistemic research integrity, and since data sharing for transparency is an important part of epistemic research integrity, funders *pro tanto* ought to promote data sharing for transparency. From a philosophical perspective, epistemic research integrity does not require data sharing for secondary use, and from a community consensus perspective it is clearly considered as part of epistemic integrity only in a few cases of specific scientific communities. Therefore, a universal obligation for funders to promote data sharing for secondary use cannot be derived from considerations of epistemic research integrity.

## Further Relevant Moral Obligations

In this section, we present two further obligations that partially speak *in favour* of funders promoting data sharing and partially *against* it. After presenting these various obligations in the following, we will close the section by weighing all pertinent obligations of funders and come to an *all things considered* judgement.

1. Funders have a *pro tanto* obligation to respect the rights of individuals and to not harm human or non-human beings, which includes the obligation to not induce, cause or increase risks of harm and of rights violations. This includes the obligation to respect privacy and informational autonomy of data subjects and not to induce, cause or increase informational risks or harms This obligation is part of the obligation to promote the socio-moral integrity of funded research and it speaks both *in favour* and *against* the promotion of data sharing:
  i) As data sharing reduces the need for ever-new data collection, data sharing also reduces the amount and frequency of research procedures in interventional and non-interventional studies that carry risks for participants (Fischer & Zigmond, 2010). Hence, in this regard the obligation to respect the rights of persons and to not harm anybody speaks in favour of funders’ promoting data sharing.
  ii) The sharing of research data and its ensuing secondary use increases informational risks for data subjects. *Prima facie*, this speaks against the promotion of data sharing. However, if subjects are informed about these risks and give consent to the usage of their data despite these risks, this increase of informational risks does not represent an infringement of the obligation not to harm. *Volenti non fit inuria*. Thus, the risks do not speak against funders promoting data sharing if consent is obtained in funded research. Of course, this argument raises the question of a model that offers research subjects appropriate information and opportunities to consent or reject consent and, at the same time, allows for data sharing without causing unreasonable practical burdens or hurdles (Manson, 2019; Mikkelsen et al., 2019; Ploug & Holm, 2016). We deem that broad consent, if combined with a normative and technical governance framework and data protection measures, is an appropriate information and consent model. In order to meet their obligation to respect the rights of data subjects, funders should thus recommend that broad consent be embedded in appropriate normative and technical governance frameworks. Irrespective of the question of informed consent, informational risks exist due to data misuse and data breaches. Erlich & Narayanan, 2014; Hayden, 2013; Homer et al., 2008; Levy et al., 2007 have shown how different techniques could be used for breaching (particularly genetic) privacy. These risks *pro tanto* speak against the promotion of the sharing of personal data.
  iii) The pooling of data from different sources and the use of big data methods enables predictions about sensitive information regarding persons or groups other than the original data subjects (Mühlhoff, 2021). Some authors warn that this increases risks of stigmatisation and discrimination of marginalised groups (Favaretto et al., 2019; Reed-Berendt et al., 2022; Xafis et al., 2019). Promoting and accelerating data sharing and secondary use expand the opportunities for pooling and big data and thus might increase these risks. Thus, in this regard the obligation to minimise risks of harm speaks against the promotion of data sharing.
2. Funders also have a *pro tanto* obligation to increase public trust in science and research funding. This obligation partly speaks in favour and partially speaks against the promotion of data sharing. On the one hand, as data sharing promotes transparency and accountability, it can increase and consolidate public trust and confidence in science and research funding. Hence, in this respect funders ought to promote data sharing in order to promote public trust. On the other hand, since promoting data sharing increases risks for privacy and creates challenges for informational self-determination, concerns about these risks and challenges might reduce trust in the research system (Platt et al., 2018; Ploug, 2020). Hence, in this respect funders ought not to promote data sharing in order to promote or maintain public trust.

However, the extent to which the two obligations (not to harm and respect rights and to foster public trust) speak *against* the promotion of data sharing can be significantly reduced. In fact, funding agencies can and should do various things to minimise or prevent the pertaining risks:

1. They should fund technological as well as ethical, legal, and social research (ELSA-research) on practical solutions for data security and privacy protection with a particular view on problems and risks resulting from big data and machine learning.
2. Funders should promote research on data augmentation and synthetic data as potential approaches to handle limitations to data sharing due to risks for data subjects.
3. They should finance and promote data infrastructures and archives or repositories that can guarantee data privacy and security and require funded researchers use these trusted repositories.
4. Funders should fund the development and implementation of data access committees that take into account the aforementioned risks resulting from secondary use.
5. Funders should support data stewardship infrastructures that convey “a fiduciary (or trust) relation” that also takes into consideration the rights of patients and participants (Rosenbaum, 2010).
6. Funders should develop principles and provide best practices that support and enable researchers to provide appropriate forms of consent with regard to data sharing. They should create a framework for protecting the privacy of research participants that provides guidance on how participant information and (broad) consent forms are to be designed.
7. Funders should provide standards and best practice for contracts between data producers, repositories, and data re-users with special attention to data protection and security.^26^
8. In all of the aforementioned measures, the participation and inclusion of patient representatives should be promoted and enabled.^27^
9. Funders should require researchers to reflect upon and identify potential risks (early in the process) by creating a data management plan, elaborating how they address and intent to deal with or avoid these risks.

If the pertaining risks are addressed and thus comparatively small, the trust obligation and the not to harm obligation rather speak in favour of the promotion of data sharing or at least have no significant weight against data sharing. Even if they keep having some limited weight against data sharing, they are outweighed by the obligations in favour of promoting data sharing, i.e., the obligations of social value and scientific progress.^28^ Of course, the more funders encourage and press researchers to share persona-related (non-anonymous) data, the more they are responsible for the impact of their policies on data subjects and the more they have to support researchers in protecting data subjects’ informational rights and privacy and this increases the financial and administrative costs and burdens for funders. However, we do not think that this outweighs the benefits in terms of social value and scientific progress.

The main conclusion of Sects. "The Moral Obligations of Funders and the Promotion of Data Sharing" and "Further Relevant Moral Obligations" is thus: Although there are two *pro tanto* obligations that speak against the promotion of data sharing by public funders, the *pro tanto* obligations in favour of the promotion weigh heavier (provided that the mentioned risk reducing measures are implemented). Public funders thus have an *all things considered* obligation to promote the sharing of data of funded researchers.^29^

## Mandatory Data Sharing Policies and Academic Freedom

Up to this point, we have not directly commented which means funders ought to use to promote data sharing. As we said, it is an empirical question what follows from the *pro tanto* obligations of funders to promote data sharing in terms of specific means to promote it.

However, in the following we want to examine a question with regard to a specific means of promoting data sharing: *Mandatory Data Sharing Policies*. Funders are increasingly advised to adopt policies that *require* data sharing (Sim et al., 2020). The NIH as a major funder has been setting standards in implementing such policies for years now and has recently implemented a new mandatory data sharing policy (Kozlov, 2022; National Institutes of Health, 2022). We find it plausible that such policies are comparatively effective and efficient. Mandatory data sharing policies can be designed at least with two different objectives: 1. They can require only the sharing of data that is the evidential basis for an already published paper and only for purposes of transparency and confirmability. 2. Or they can additionally require the sharing of data (either publication-related or all data that are generated during a research project) for purposes of secondary use.

As mandatory data sharing policies of public funders restrict the individual freedom of funded researchers (at least if these are dependent on third-party funding), the question arises whether such policies conflict with *academic freedom*. Do data sharing requirements implemented by public funders infringe on the academic freedom of individual researchers?^30^

To answer this question, we have to clarify what academic freedom is and what it protects. From a philosophical perspective,^31^ academic freedom is first and foremost the *negative right* of individual researchers against external intervention in their scientific work and decision-making. Academic freedom mainly concerns the freedom to choose research questions, theories, and methodologies as well as publication venues independently from outside intervention, *in particular state intervention*. This *negative right* of freedom from intervention of researchers thus corresponds to the *negative duty* of the state not to intervene.

As public funders are (semi-)governmental institutions whose funding comes predominantly from government budgets and on whose boards government representatives participate in decision-making, the following holds: Public funders have the negative obligation to respect the negative right to academic freedom of researchers.

The question now is whether mandatory data sharing policies violate the negative right of researchers to academic freedom? To answer this question, we must determine in more detail the scope of protection of academic freedom. From our perspective, the scope of protection of academic freedom includes only actions of researchers that are not violations of crucial and basic norms of epistemic research integrity. Such crucial and basic norms determine fundamental requirements of science and research as a specific kind of rational practice and communication. For instance, researchers that engage in data fabrication or falsification fail to meet such fundamental requirements. They thus violate crucial and basic norms of research integrity and engage in behaviour that is not protected by academic freedom.

Hence, we must answer the following questions:

1. Is the omission (or refusal) to share data that are the evidential basis of published research results for purposes of transparency and reproducibility a violation of fundamental requirements of scientific work and communication?
2. Is the omission (or refusal) to share data (either publication-related or all data that are generated during a research project) for purposes of secondary use a failure to meet such fundamental requirements?

**Ad 1**: We believe that not sharing data that underlies research results (a published paper) for purposes of transparency is a violation of the fundamental requirements of scientific work and communication. This is clearly the case from the philosophical perspective on research integrity. Although not sharing data that underlies a published paper for transparency seems to be a less severe scientific misconduct as data fabrication or falsification, it clearly runs counter to one of the basic requirements of scientific communication and (collective) truth-seeking: To make one’s own scientific work transparent and reproducible. There is no reasonable justification for why researchers should be generally free to avoid that their published (!) work can be reviewed in all its parts.^32^ We believe the philosophical perspective is backed by the perspective of the consensus of the scientific community. The community recognises data sharing for transparency as a key requirement of epistemic research integrity. Almost all codes of conduct and guidelines on research integrity emphasise the close relation between honesty, reproducibility, and data transparency (see Sect. "The Obligation to Promote the Epistemic Integrity of Research". A). Therefore, research without sharing data for transparency *is not protected* by academic freedom. Thus, mandatory policies that require data sharing for transparency *do not infringe* on the right to academic freedom of individual researchers.

**Ad 2**: In Sect. "The Obligation to Promote the Epistemic Integrity of Research", we have already noted that neither in the *community consensus* nor in the *normative-philosophical* perspective data sharing for secondary use is a requirement of epistemic integrity. This means that the freedom to share or not to share data for secondary use is within the scope of protection of academic freedom. However, data sharing requirements for secondary use of public funders are not necessarily an infringement of academic freedom. First, it depends on how much researchers must rely on third-party funding in their research. If they have access to basic financial resources of their institutions and are not dependent on applying for additional public funding, then such requirements are not a restriction of their academic freedom. Second, data sharing requirements of public funders that enjoy relative autonomy from government and whose decisions are essentially made by scientists themselves do not represent state coercion but rather self-determination of the scientific community. However, academic freedom does not only protect individuals against state intervention but also against infringements through (parts of) the scientific community. Thus, data sharing requirements of public funders with state autonomy (and also those without state autonomy) do represent an infringement of academic freedom (at least for researchers that depend on their funding), though not in the classical sense of state infringements.

It must be noted, however, that this infringement of academic freedom is a fairly *small* one. The freedom to share or not to share data for secondary use does not belong to the core of academic freedom. The core arguably is the freedom to “follow a line of research where it leads” (Russell, 1993), i.e., the freedom to choose research questions, theories, and methodologies as well as publication venues independently from outside intervention. Nonetheless, it *is* an infringement, but we believe it can be mitigated by the following measures: Funders can a) offer the possibility for a justified exception from data sharing requirements (for instance, for reasons of data protection or dual use risks), b) allow for an embargo period in which the funded and data producing researcher has the exclusive privilege to use their data, c) consider discipline-specific standards for data management and sharing, and d) compensate for burden and costs financially (for instance, for fees of repositories for long-term storage or for data protection measures) and through investments in and supply of technical and administrative support (for instance, digital privacy and security safeguarding solutions and best practices). If funders implement measures like these, the infringement of academic freedom through mandatory data sharing policies becomes so small that it can be justified with reference to the other *pro tanto* obligations of funders, namely the obligations with respect to social value, scientific progress, and the minimisation of harm.^33^

However, the justifiability of the infringement on academic freedom through mandatory data sharing policies is dependent on a further condition: Mandatory policies can only be justified, if there are no measures of promoting data sharing that are more effective and less invasive in terms of academic freedom.

A last word on the implications of the diagnosis that policies that require data sharing for secondary use infringe on the academic freedom of researchers: If public funders infringe on the academic freedom of researchers with reference to the benefits of data sharing, they have the responsibility to ensure that these benefits are realised. This requires two things of them: 1. Since the benefits of data sharing only materialise if reproduction and replication as well as secondary use are actually carried out, funders should fund appropriate projects. They should finance and reward reproduction and replication studies and set up a funding programme for secondary research. 2. Funders should fund research and monitoring on whether their own initiatives to promote data sharing are i) effective in terms of actual data sharing and ii) actually lead to the hoped-for benefits.

## Summary and Conclusion

In this paper, we investigated the question whether public funders have a moral obligation to promote the sharing of research data generated in funded research projects. More specifically, we asked which of funders general moral obligations speak in favour of and which of these obligations speak against the promotion of data sharing. We draw the following conclusions: First, public funders have several general *pro tanto* obligations that (under certain conditions) require funders to promote data sharing. The main obligations are the social value-, scientific progress- and epistemic research integrity-obligation. Second, in the assessment of *pro tanto* obligations against promoting data sharing, we argued that – provided that funders take measures to minimise the risks for research subjects and third parties – the obligations in favour of promoting data sharing outweigh the obligations against. Therefore, we concluded with respect to our overall research question that public funders ought to promote data sharing *all things considered*.

With respect to our third specific research question whether mandatory data sharing policies are an ethically justifiable means of promoting data sharing, we argued: First, the scope of protection of academic freedom does not cover the omission or refusal to share data for purposes of transparency. Requirements to share data for the purpose of transparency therefore do not violate academic freedom. Second, the scope of protection does cover the omission or refusal to share data for secondary use, therefore requirements to share data for secondary use violate academic freedom to a small extent (at least for researchers that are dependent on public funding). However, such requirements and thus the violation of academic freedom can be justified with reference to the other *pro tanto* obligations that public funders have.

## Footnotes

1 Sometimes research data can only be re-used when research methodologies that have been used to collect, generate and analyse the data (questionnaires, analytical codes, etc.) are shared as well (Goldacre et al., 2019). Thus, sharing these methodologies and other intermediary resources might equally be important as sharing the data themselves. However, due to some disanalogies between data and those resources (most saliently the fact that some of the latter can be seen as intellectual property), we confine our discussion here to research data.

2 The Creative Commons set of licenses are the most commonly used for sharing research data. These licenses are designed to be open, which means that data can be freely reused without requiring explicit permission as long as the terms of the license are adhered to. Such licences can be a good and efficient way of reducing costs and burdens for data sharing, while they may have limited applicability in cases of person-related data or for researchers who wish to retain control over the subsequent use of the data they produce.

3 If regulatory considerations limit the sharing of data generally or on an international level, the generation of synthetic data can be an alternative. However, (sharing of) synthetic data can only complement but not fully replace (the sharing of) non-synthetic data.

4 *Pro tanto* obligations are what David Ross (1930) called *prima facie* obligations. In line with the established terminology “*pro tanto* and *all things considered* moral reasons” (Alvarez, 2016), we chose to deviate from Ross’ terminology, see also Hurtig (2007) and Dabbagh (2018).

5 Similar claims are made with regard to journal policies in Federer et al. (2018) and Gabelica et al. (2019).

6 For the debate about the social value requirement, see Barsdorf and Millum (2017), Pierson and Millum (2018), Resnik (2018a, 2018b), Wendler and Rid (2017), Wertheimer (2015).

7 See also Bierer et al. (2018).

8 For further discussions on this topic see Winkler et al. (2023).

9 Notice that the references only state that research must have *sufficient* benefits for society in order to be justified if it exposes participants to risks. However, we find this implausible and believe that it has to maximise benefits. For it seems questionable to choose project A over the alternatively fundable projects B and C, if it can be expected that either project B or C have more social benefit than A.

10 Notice that this obligation does not require a short-sighted restriction to immediate benefits and “mere” application-oriented research but will plausibly take into account basic research that enables long-term fruitful and sustainable research by exploring fundamental causal mechanisms. Otherwise, maximisation would hardly be possible.

11 These conditions also secure that data sharing of funded projects does not facilitate the exploitation or extraction of resources from the underprivileged to the privileged or to private corporations and does not promote epistemically biased research. See Leonelli (2023) for examples of such detrimental effects of data sharing.

12 This issue of the cost–benefit balance of promoting data sharing is also pertinent for all other obligations we will discuss below. We will not mention it again though and assume for the rest of the paper that the benefits of promoting data sharing are greater than the costs.

13 Bierer et al. (2018) also argue that funders ought to promote data sharing in order to advance the social value of research. Notice that this obligation might be stronger or weaker for particular research fields or specific data. For instance, the social value of sharing particular health data in a pandemic or biomedical data in general is presumably bigger than the social value of the sharing of archaeological data about a particular Egyptian pharaoh.

14 We believe that on any other plausible account of social value, i.e., on any plausible distributive principle funders ought to promote data sharing *and* fund research that has social value. For instance, a utilitarian account of social value will give us the same conclusion.

15 On the notion of scientific progress and “significant” knowledge, see Bird (2007), Kitcher (2001), Niiniluoto (2019).

16 For the view that scientific knowledge has intrinsic value, see for instance Schwartz (2020).

17 What holds for the social value obligation also holds for the obligation to promote scientific progress: Depending on the particular research field and the particular data the obligation to promote data sharing in order to promote scientific progress is stronger or weaker. The sharing of particular (kinds of) data might bear more potential to promote scientific progress while the sharing of other (kinds of) data might bear less potential.

18 For different terminologies for both kinds of obligations, for instance internal vs. external norms, see Resnik (1996) and Reydon (2013). For an attempt to differentiate the justificatory grounds for the various kinds of obligations of scientists see Resnik (1998).

19 For a legal analysis of the relation between (semi-)governmental promotion of data sharing and good scientific practice in the context of German constitutional law see Fehling and Tormin (2021).

20 Strictly speaking, evidence is that which confirms or disconfirms a scientific claim, i.e., data. A methodology or an analysis is not evidence in this sense. However, we stick to the understanding of Munafò above because the sharing of evidence in his sense is required by the norm of evidence-sharing. At least we think that Haack has this in mind.

21 Robert Merton (1942/1973) famously introduced this term in his description of “the normative structure” and the “ethos of science”, see also Ziman (2009).

22 Although Fanelli (2018) doubts that misconduct has a major impact on the scientific literature, she agrees that it is non-negligible.

23 For instance, Tsuyoshi Miyakawa (2020) reports the results of analyses on the manuscripts that he has handled as Editor-in-Chief of *Molecular Brain* as showing that “more than 97% of the 41 manuscripts did not present the raw data supporting their results when requested by an editor, suggesting a possibility that the raw data did not exist from the beginning, at least in some portions of these cases”.

24 The DFG Guideline (2019) is arguably a guideline exclusively for *epistemic* research integrity, and it is thus reasonable to assume that the explicit inclusion of data sharing for secondary use means that it is considered to be an *epistemically* required practice. However, the ALLEA code (ALLEA 2017), as some other codes, is not exclusively focused on epistemic integrity as it includes socio-moral obligations (for instance, to respect the needs and right of study participants). Its statement that data should be as open as possible, as closed as necessary can be understood as including data sharing for secondary use, but it remains open whether this is taken to be a requirement of *epistemic* integrity. It could be case that the justification for data sharing for secondary use is mainly seen in its benefit for society and scientific progress. *If* this is the reason why data sharing for secondary use is included in research integrity, then the research integrity obligation adds nothing to the social value and scientific progress obligation with respect to data sharing for secondary use – which we already discussed in Sect. "The Obligation to Benefit Society" and "The Obligation to Promote Scientific Progress".

25 Only in cases in which there are strong philosophical or ethical reasons that speak against the community consensus, funders might not be allowed to follow this consensus. However, we believe this is not the case for the issue of data sharing for secondary use.

26 There have been intense and broad research and debates on ethical, legal, and social issues of privacy and data protection and other informational aspects of research subject protection in biomedical data intense research and data sharing for 10–15 years. Following the increasing activities of genomic data sharing, approaches and best practices have been developed to address challenges concerning data protection, privacy, and informational rights and autonomy. See for instance the GA4GH and its “Regulatory and Ethics Work Stream” (https://www.ga4gh.org/how-we-work/workstreams/) that provides standard solutions for genetic data sharing and a framework for responsible sharing of genomic and health-related data (https://www.ga4gh.org/genomic-data-toolkit/regulatory-ethics-toolkit/framework-for-responsible-sharing-of-genomic-and-health-related-data/) or the European Genome Archive (EGA) which also provides best practices for genetic data sharing.

27 We develop a systematic approach to funders' responsibilities for the protection and participation of data subjects from a legal and ethical perspective in Fehling et al. (2023).

28 Since the pertaining risks are mainly associated with data sharing for secondary use, and since data sharing for secondary use is not a requirement of research integrity, the weighing of obligations here must exclude the obligation to promote research integrity and focus only on scientific progress and social value.

29 Of course, we cannot exclude the possibility of very specific cases in certain areas of research where there are additional reasons against the promotion of data sharing which override the *pro tanto* obligation that speak in favour of promoting data sharing. For example, sharing huge amounts of high quality data used to develop machine learning programs in biomedicine with a Russian research institute closely linked to the Russian military complex might bear the risk of harmful consequences for society. Our *all things considered* claim should thus be understood as not applying to such special cases. For the possibility of such cases see footnote 11 and the reference to Leonelli (2023).

30 How differently the relation between academic freedom and data sharing requirements is perceived by German funders as compared to non-German funders is examined in more detail in Anger et al. (2024).

31 The following is a philosophical and not a legal analysis. For a legal analysis of the possibilities and limits of (semi-)governmental promotion of data sharing in the German context see Overkamp and Tormin (2022) and for the German and European context with a side glance at US constitutional law see Fehling and Tormin (2021).

32 Of course, there can be specific reasons in a particular case not to make data transparent for confirmation efforts (such as, for instance, privacy concerns). However, our point is that besides such special circumstances, there is no reason for why researchers ought to generally be free to refuse to make their data available for confirmation.

33 Of course, this depends on how strong these *pro tanto* obligations are with respect to particular (kinds of) data. As we explained in footnotes 13 and 17 the weight of these obligations depends on how much the sharing of particular data from a particular research field contributes to social value and scientific progress. We believe, however, that for the most part of the sciences the sharing of research data is so valuable in theses respects that an infringement of academic freedom can be justified.
